# Habitat Heterogeneity and Connectivity: Effects on the Planktonic Protist Community Structure at Two Adjacent Coastal Sites (the Lagoon and the Gulf of Venice, Northern Adriatic Sea, Italy) Revealed by Metabarcoding

**DOI:** 10.3389/fmicb.2019.02736

**Published:** 2019-11-26

**Authors:** Simona Armeli Minicante, Roberta Piredda, Grazia Marina Quero, Stefania Finotto, Fabrizio Bernardi Aubry, Mauro Bastianini, Alessandra Pugnetti, Adriana Zingone

**Affiliations:** ^1^Institute of Marine Sciences, National Research Council, Venice, Italy; ^2^Department of Integrative Marine Ecology, Stazione Zoologica Anton Dohrn, Naples, Italy

**Keywords:** 18S rRNA gene, marine protists, northern Adriatic Sea, functional diversity, protist community structure

## Abstract

The Lagoon of Venice (LoV) and the Gulf of Venice (GoV), two adjacent coastal Long Term Ecological Research (LTER) sites in the northern Adriatic Sea, represent a transitional/marine coupled ecosystem under the influence of regional and local factors. In this study, these sites were sampled on four dates from April 2016 to February 2017 for environmental DNA and relevant abiotic variables, aiming to assess the relative importance of habitat heterogeneity and connectivity in structuring the protist community. High Throughput Sequencing of V4-18S rRNA gene from 56 samples collected at seven stations produced ca 6 million reads, grouped into 7,336 Operational Taxonomic Units (OTUs) at 97% similarity, which were affiliated to protists belonging to 34 taxonomic groups. The whole community was dominated by Bacillariophyta, especially in spring-summer in the LoV, and by Dinophyta, mainly in the GoV. Ciliophora, Syndiniales, and Cryptophyceae were the next more abundant groups. The community structure varied across the seasons and was different in the two ecosystems, which shared 96% of the reads but showed a high proportion of OTUs distributed preferentially in one of the two sites (specialists) and a different partitioning of trophic categories. GoV specialists were mainly Dinophyceae (>56%), followed by Syndiniales and Bacillariophyta, while the LoV specialists were distributed among several groups, including Bacillariophyta, Syndiniales, Ciliophora, Cryptophyceae, and Trebouxiophyceae. The main abiotic drivers of the differences between protist communities were salinity and temperature, which however explained a minor part of the variance (17%), pointing at a higher relevance of biotic factors and inter-taxa relationships. This was more evident in the LoV, where the network analysis highlighted a higher number of OTUs’ connections than in the GoV. Overall, the metabarcoding approach allowed to depict the composition of the whole protist community in the lagoon and adjacent coastal waters with high resolution, revealing many taxa so far not reported in the area. In addition, despite no clear barrier to dispersal processes, differences in the relative abundance and temporal variability of local protist communities indicate that environmental heterogeneity, in these adjacent and connected ecosystems, can be strong enough to allow for ecological segregation.

## Introduction

Microbial eukaryotes are versatile components of aquatic environments, covering multiple functional roles – from autotrophy to heterotrophy (predators, decomposers, parasites) and mixotrophy – and contributing to biogeochemical cycling (Worden et al., 2015; Keeling and del Campo, 2017). Unveiling the diversity of microeukaryote communities contributes remarkably to our understanding of microbial food web structure in aquatic ecosystems. For a long time, this pivotal component of the marine environment could only be explored morphologically, with resolution levels that left most small and featureless forms largely unexplored. Recent technological developments of molecular microbial ecology have expanded our capacity to describe and investigate the community diversity and structure and the biogeography of the single-celled eukaryotes, informally called protists (Caron et al., 2012; Leray and Knowlton, 2016).

These molecular approaches have been widely applied in protist studies in different aquatic ecosystems, including inland, oceanic and coastal waters, and extreme environments (e.g., de Vargas et al., 2015; Ainsworth et al., 2017; Zhang et al., 2017; Brannock et al., 2018). Community composition of planktonic protists may differ among offshore (e.g., de Vargas et al., 2015; Malviya et al., 2016), coastal regions (e.g., Bittner et al., 2013; Massana et al., 2015) and water depths (e.g., Pawlowski et al., 2011); further the community may shift over seasons as well as over smaller (days to weeks) time scales (e.g., Berdjeb et al., 2018; Nagarkar et al., 2018).

In ecological studies of protistan communities, one key goal is understanding the patterns of community biodiversity and structure, at both the spatial (Massana et al., 2015; Logares et al., 2018; Das et al., 2019) and temporal scales (Genitsaris et al., 2015; Berdjeb et al., 2018), which in turn requires investigation of the factors that shape the communities (Steele and Ruzicka, 2011; Wang et al., 2013; Monard et al., 2016; Xue et al., 2018).

Combining molecular data of marine microeukaryote communities with tools from macroecology can be particularly useful to investigate the main factors affecting the community structure and patterns. In coupled ecosystems, such as transitional waters and the adjacent marine environment, habitat heterogeneity, and connection coexist as intrinsic ecological features. Transitional water ecosystems (Eureopean Union, 2000; Elliott and McLusky, 2002), such as shallow coastal lagoons, are fairly heterogeneous, mainly due to the geomorphology and catchment geology, the close benthic–pelagic coupling, the freshwater inputs, and the marine water exchanges – through tides and currents – with the adjacent marine ecosystems (Basset et al., 2006; McLusky and Elliott, 2007; Vadrucci et al., 2007). Through the connection between transitional and marine waters, complex coupled ecosystems are established, where the regional and local factors are at play in shaping the planktonic community structure.

In the northern Adriatic Sea, the Lagoon of Venice (LoV), and the Gulf of Venice (GoV) are examples of such transitional/marine coupled ecosystems. Both are research sites belonging to the Long Term Ecological Research (LTER) network LTER-Italy^1^. LTER is an essential component of worldwide efforts to improve our knowledge of the structure and functions of ecosystems and of their long-term response to environmental, societal and economic drivers (Mirtl et al., 2018). LTER is organized in distributed networks of research sites at the global (ILTER, LTER-International), regional (LTER-Europe) and national levels. LTER-Italy, a formal component of ILTER and LTER Europe, consists of 79 research sites, which include terrestrial, freshwater, transitional and marine ecosystems (Bergami et al., 2018). The marine component of LTER-Italy is mainly represented by transitional and coastal ecosystems (Pugnetti et al., 2013).

Therefore, consistent knowledge exists on the LoV and the GoV, in particular on phytoplankton communities and related abiotic factors (Acri et al., 2004; Bernardi Aubry et al., 2004, 2012, 2013; Facca et al., 2014), which may provide a useful background for molecular investigation on the whole protist community.

In the area, a High Throughput Sequencing metabarcoding approach has so far been applied to the bacterial communities (Quero et al., 2017), while a few protist samples collected in June 2016 have been analyzed in the frame of the Ocean Sampling Day (OSD) project (e.g., Penna et al., 2017; Tragin et al., 2018; Tragin and Vaulot, 2019). In this study we analyze for the first time in the area the entire microeukaryote community through a metabarcoding approach, by sequencing the V4 region of the 18S rRNA gene. We analyse protist communities sampled at different temporal (seasonal) and spatial (lagoon and sea) scales, aiming at contributing to the identification of the relative importance of regional processes and local characteristics in structuring the protist community in aquatic ecosystems. Our study focuses on the differences between the lagoon and sea communities, with insights into their functional diversity revealed by their trophic habits and on the affinity of individual taxa for each of the two environments investigated.

## Materials and Methods

### Study Area and Sampling Strategy

The LoV, the largest (550 km^2^) lagoon of Italy and one of the largest in the Mediterranean basin, is a microtidal, polyhaline lagoon, located in the north-western part of the Adriatic Sea in a densely inhabited and industrial area, affected by high numbers of tourists and hosting ports, shipyards, marinas, fisheries, and aquaculture (Viaroli et al., 2007; Solidoro et al., 2010). The LoV has an average depth of 1 m with large shallow areas and a network of deeper (5–10 m) navigation channels. Twelve main tributaries discharge an annual average of about 35 m^3^ s^–1^ of freshwater into the lagoon, with seasonal peaks in spring and autumn (Solidoro et al., 2010). The LoV is connected to the coastal waters of the GoV through three inlets (Lido, Malamocco, and Chioggia), with water exchanges mainly governed by a microtidal (average amplitude 100 cm) regime (Solidoro et al., 2010). Water residence time ranges from a few days (close to the inlets) to 1 month (in landward areas), depending on the interplay of tide, wind, and topography (Umgiesser et al., 2014; Ghezzo et al., 2015).

The GoV is a shallow marine system (maximum depth: 45 m) that receives the freshwater and nutrient inputs of several rivers, of which the Po (the main Italian river) is the major contributor, while in its eastern part it is influenced by the more saline and oligotrophic waters from the southern Adriatic basin. As a consequence, the trophic conditions are remarkably variable at both the spatial and temporal scales, ranging from the permanently meso-eutrophic western coastal area to the highly dynamic transition zone with saline oligotrophic waters offshore (Franco and Michelato, 1992; Socal et al., 2008).

**FIGURE 1:**
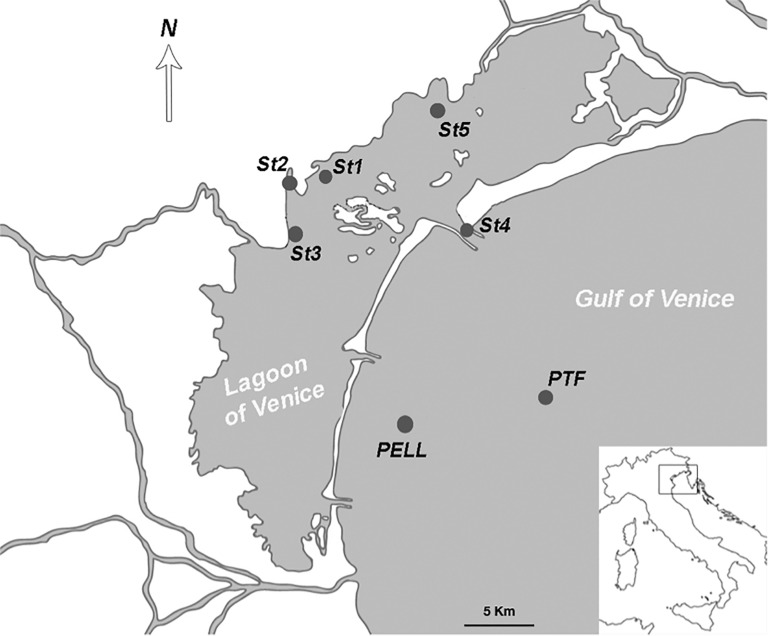
The study area with the seven sampling stations.

The area considered in this study comprises the northern and central part of the LoV, and the coastal area from the Lido inlet up to 15 km offshore (Figure 1). Four sampling campaigns, in April, July, November 2016, and February 2017 were conducted at seven stations. Four stations (St1, St2, St3, and St5) were located in the LoV and three in the coastal areas of the GoV (St4, PELL, and PTF); six of them (St1, St2, St3, St4, St5, and PTF) are LTER stations. The stations in the LoV were chosen more than 20 years ago as representative of the natural and anthropogenic environmental variability of the northern and central basins, being influenced by a complex interplay of freshwater and marine inputs (Solidoro et al., 2004; Cucco and Umgiesser, 2006) and by different human impacts (Bianchi et al., 2003). Stations St4 is located in one of the three inlets, while PELL and PTF are in the coastal and offshore GoV, respectively, with PTF close to the oceanographic tower “Acqua Alta”^2^.

### Environmental Parameters

The four sampling campaigns were performed separately for each environment in two consecutive days, always at neap tide, sampling only the near-surface water layer. At each station, temperature and salinity were measured with a CTD SBE 911 and water samples were collected with a Niskin bottle.

For chlorophyll *a* (Chl *a*), 2 L of seawater from the GoV or 500 mL from the LoV were immediately filtered through Whatman GF/F fiberglass filters (nominal porosity = 0.7 μm); filters were stored frozen and subsequently analyzed according to Holm-Hansen et al. (1965). Dissolved Inorganic Nitrogen (DIN: sum of ammonia, nitrite, and nitrate), orthophosphates (P-PO_4_) and orthosilicates (Si-SiO_4_) in filtered seawater were analyzed with a Flow-Solution III autoanalyzer (OI-Analytical) Systea-Alliance auto-analyser, according to Strickland and Parsons (1972) and Hansen and Koroleff (1999).

### Filtration, DNA Extraction, and Sequencing

At each of the seven stations on the four sampling dates, water samples (3 L) were collected in duplicate in the near-surface layer, prefiltered on a 200 μm mesh-size net and then filtered onto cellulose ester 1.2 μm pore size filters (47 mm Ø, Whatman) using a peristaltic pump. A total of 56 filters were obtained and stored at −80°C until molecular analysis. Total DNA from each filter was extracted using the DNeasy 96 Plant Kit (QIAGEN) according to the manufacturer’s instructions. DNA concentrations were determined by Qubit dsDNA HS kit (Thermofisher) and the DNA samples were stored at –80°C until PCR. The hypervariable V4 region of eukaryote SSU rRNA gene was amplified using the primers TAReuk454FWD1 and TAReukREV3 (Stoeck et al., 2010) modified as in Piredda et al. (2017) (5′ CCAGCASCYGCGGTAATTCC-3′ 5′A CTTTCGTTCTTGATYRATGA-3′). Finally, the hypervariable V4 region was sequenced (2 × 250 bp sequencing) on the Illumina MiSeq platform as described in Piredda et al. (2017). Raw sequences were deposited in the Sequence Read Archive (SRA)^3^ under the BioProject PRJNA576330^4^.

### Sequence Analyses

Paired-end reads were processed using Mothur v.1.33.0 (Schloss et al., 2009). Contigs between read pairs were assembled and differences in base calls in the overlapping region were solved using ΔQ parameter as described in Kozich et al. (2013). Primer sequences were removed (pdiffs = 3), and no ambiguous bases were allowed; the maximum homopolymer size was 8 bp. The remaining sequences were dereplicated and screened for chimeras using UCHIME in *de novo* mode (Edgar et al., 2011). Taxonomic assignment was initially performed using a naïve Bayesian classifier (Wang et al., 2007) trained using the PR2 database (v.4.10.0^5^; Guillou et al., 2013), with an 80% bootstrap confidence threshold, in order to detect non-protist groups (including Bacteria, Archaea, Metazoa macroalgae and Fungi), which were excluded from further analyses. Sequences were clustered into operational taxonomic units (OTUs) at 97% of similarity using vsearch (Rognes et al., 2016) clustering (method = dgc) through Mothur. OTUs containing only one read (singleton) were removed from downstream analyses. Taxonomic assignment was performed on a single representative sequence from each OTU (the most abundant) using BLASTN (Altschul et al., 1990) against the PR2 database [v.4.10.0 (see footnote 5); Guillou et al., 2013], discarding the assignments with similarity ≤ 90% and query coverage ≤ 70% of the sequence length. Based on these criteria, 1.5% of the sequences were excluded from further analyses.

### Functional Diversity

To gain insight into different trophic levels of protists, taxa identified as described above were classified into four trophic functional groups: autotrophs, heterotrophs, mixotrophs, and parasites, based on information from the literature (Tomas and Hasle, 1997; Mangot et al., 2011; Not et al., 2012; Klais et al., 2017, and other specific papers in Ramond et al., 2018). The four selected trophic categories were sufficient for the identification of similarities and differences between the two environments investigated, while a functional investigation based on more detailed annotations of nutritional modes is beyond the aim of this study. Assignment to different categories was made at the group level in some cases, but the individual species, genera (or families) were considered whenever possible. We conventionally attributed only Bacillariophyta and Mamiellophyceae to autotrophs, whereas all other chloroplast-bearing taxa were considered as mixotrophs. Some groups as Labyrinthulea and Oomycota, which can be parasites, commensalists and/or saprophytes, were all included in parasites, as any other separate categories would consist of a low number of OTUs. Some unresolved supraspecific taxa (e.g., *Gymnodinium* spp.) or unnamed species (e.g., Dinophyceae_XXX) were not attributed to any specific trophic group in case they could include both autotrophs and heterotrophs. These taxa were annotated as “information not available (NA).”

### Statistical Analyses

Hierarchical clustering of the 56 samples based on Bray–Curtis distance matrix showed duplicates for each sampling event to be paired in almost all cases (data not shown). The duplicates were hence pooled, resulting in a dataset of 28 samples. All the statistical analyses and plots were generated using several R packages (R version 3.5.2; R Core Team, 2014). Rarefaction curves of observed OTUs and α-diversity estimators (Shannon index, H) were calculated on this total dataset. For multivariate and comparative analyses, the abundance dataset was normalized with a random subsampling to the second lowest number of sequences (*n* = 21,181) with the “rrarefy” function, R package *vegan* version 2.5-5 (Oksanen et al., 2019). Non-metric multiDimensional Scaling (nMDS) was performed using the “metaMDS” function based on a Bray–Curtis dissimilarity matrix, using an OTU table modified following the Hellinger transformation. The adonis approach was applied to identify the explanatory environmental variables, i.e., those showing significant correlations with the community dissimilarity (Bray–Curtis index) matrix. The selected variables were used to perform Canonical Correspondence Analysis (CCA). Venn diagrams were calculated and plotted using *venn* version 1.7 R package. To assess the species’ affinities for different habitats, OTUs were classified as “generalists” and “specialists” using the “clamtest” function in the *vegan* package. Based on the abundance in two habitats and a specialization threshold (*K*), the clam multinomial model classifies taxa into one of four groups: (1) “generalist”; (2) “habitat A [lagoon] specialist”; (3) “habitat B [sea] specialist”; and (4) “too rare to classify” with confidence. As *K* we applied a conservative threshold based on the super-majority rule (*K* = 2/3, *P* = 0.005). This approach permits a robust statistical evaluation of habitat specialization for a large number of species and does not rely on measurements of individual performance or exclude rare species *a priori* (Chazdon et al., 2011). The test was performed using a sub-dataset from two marine (PELL and PTF) and at two lagoon stations (St2 and St5), selected as representative of the natural (PTF and St5) and anthropogenic (PELL and St2) variability of the two environments (Bernardi Aubry et al., 2004, 2013). For each habitat the original data from eight samples were pooled and the two resulting datasets were normalized to the lowest number (1,382,776 reads) (Supplementary Material 3, clam dataset).

For network analysis, statistical relationships between taxa were calculated as described in Jeffries et al. (2016) and Quero et al. (2017), based on the MIC-MINE (Maximal Information Coefficient – Maximal Information-based Non-parametric Exploration) algorithm (Reshef et al., 2011). For this analysis we used the whole dataset (28 samples) normalized as described above for multivariate and comparative analyses. In order to satisfy the computational requirements of MIC-MINE, the OTUs with total abundance less than 50 reads were excluded from the analysis. After calculation of MIC MINE on the OTU table, we selected only those relationships that resulted statistically significant (*p* < 0.01) upon comparison with *P*-value Tables available online^6^. The MIC value is a statistical measure that indicates the strength of the interaction between OTUs but does not provide information on their sign (positive or negative). Connections were assigned as positive or negative using the MIC-p2 (non-linearity) parameter, provided in the MIC output, as described in Hernández-Ruiz et al. (2018). MIC-p2 > 0.5, indicating non-linear relationships, were considered negative connections. Significant values were selected and analyzed in Cytoscape 3.6.0 (Shannon et al., 2003). The original network was compared against 100 randomized versions generated with Network Randomizer 1.1.2 (Tosadori et al., 2016). The NetworkAnalyzer tool within Cytoscape was used to calculate network topological parameters including the degree as a proxy for node importance (number of edges/connections arriving to or leaving from a node). The most connected nodes (high degree, range values 39–20) were considered as hub nodes. We interpreted the topology and hub nodes of the network using the classification resulting from clam test. The final visualization of the network was performed using the Force Atlas algorithm in Gephi software (Bastian et al., 2009).

## Results

### Environmental Variables

The environmental conditions widely varied across the study area and the seasons (Supplementary Table 1). Water temperature reached the minimum in February (9.0°C in the LoV and 12.9°C in the GoV) and peaked in July (24.3°C in the LoV, and 25.7°C in the GoV). Salinity was lower in the LoV (28.8–29.7) than in the GoV (33.5–34.8). Inorganic nutrient concentrations were generally higher in the LoV (DIN: 18.7–43.7 μM; P-PO_4_: 0.5–1.1 μM; Si-SiO_4_: 17.8–38.7 μM) than in the GoV (DIN: 5.4–21.8 μM; P-PO_4_: 0.2–0.3 μM; Si-SiO_4_: 5.5–14.6 μM), with peaks in November in both sites. Chl *a* fluctuated between 0.5 and 4.8 μg L^–1^ in the LoV, with peak values in July, and from 1.1 and 4.0 μg L^–1^ in the GoV, with peak values in April. Differences between the two environments were only significant for salinity and P-PO_4_ (*p* < 0.01) and for DIN and Si-SiO_4_ (*p* < 0.05). The range values recorded in the study period are quite typical for the two areas and in line with the seasonal values recorded during 5 years of time series for each area (Supplementary Table 1).

### Protist Community

#### Protist Diversity and Seasonality

The 28 samples consisted of 12,268,180 raw reads. Filtering procedure generated a final curated dataset including 5,883,770 V4-18S protist reads which were clustered into OTUs at 97% similarity. After the removal of singleton, 7,336 OTUs remained. Most of the reads (88%, 1651 OTUs) were assigned to references of named species with similarity > 99%, while for only 9% (3748 OTUs) and 3% (1937 OTUs) of the reads the similarity to a reference was in the range 98–95 and 94–90%, respectively (Supplementary Material 1, taxonomic assignations summary). Rarefaction curves on the whole dataset showed that the sequencing effort applied was sufficient to describe the protist diversity in the two areas, with an overall higher number of reads in the lagoon than in the marine samples, as well as at individual stations (Supplementary Figure 1).

**FIGURE 2:**
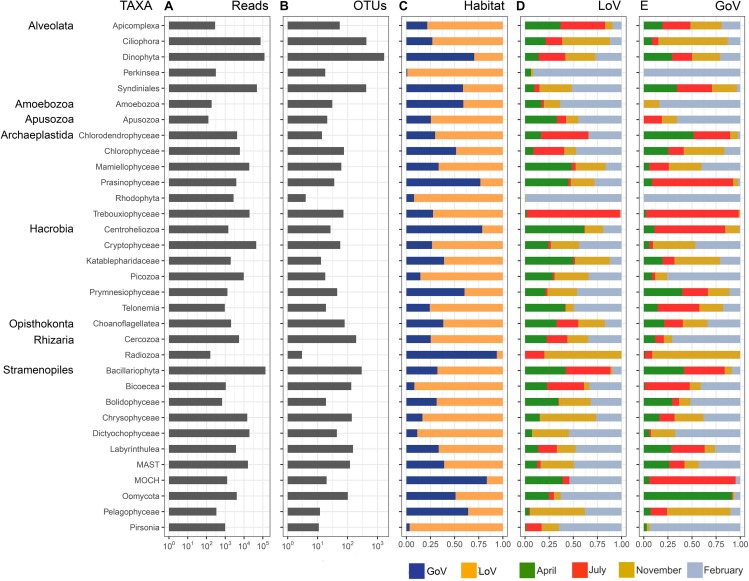
Taxonomic group composition based on the normalized dataset: **(A)** number of reads; **(B)** number of OTUs; **(C)** relative abundance of reads in the two habitats; **(D)** relative abundance of reads in the months sampled in the Lagoon (LoV), and **(E)** in the Gulf of Venice (GoV). MAST, Marine Stramenopiles; MOCH, Marine Ochrophyta.

The normalized dataset used for most analyses resulted in an overall number of 570,936 reads, corresponding to 4,506 OTUs that belonged to 34 high-level taxonomic groups (Supplementary Figure 2 and Figure 2A). Bacillariophyta and Dinophyta were the most abundant groups in the entire dataset in terms of number of reads (23.9 and 21.2%, respectively), followed by Ciliophora (13.2%), Syndiniales (8.4%), and Cryptophyceae (7.7%). Chrysophyceae, Dictyochophyceae, Mamiellophyceae, Trebouxiophyceae, MAST (nanoheterotrophic marine stramenopiles), Picozoa, Rhodophyta, and Chlorophyceae ranged between 3 and 1%. The remaining 21 taxonomic groups showed a read percentage < 1%. In terms of OTUs (Figure 2B), Dinophyta showed the highest number (1673), followed by Ciliophora (426), Syndiniales (419), and Bacillariophyta (297). Cercozoa, Labyrinthulea, Chrysophyceae, Bicoecea, MAST, and Oomycota were present with 100–200 OTUs, and the remaining groups with less than 100 OTUs.

**FIGURE 3:**
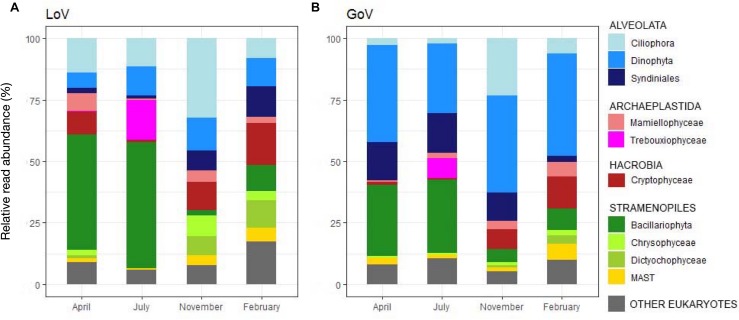
Temporal variations in the main groups of the protist communities in **(A)** the Lagoon (LoV) and **(B)** in the Gulf of Venice (GoV) based on the whole normalized dataset. Groups with read abundance < 5% are lumped in “Other Eukaryotes.”

Most groups showed different distributions and composition across sites and seasons (Figures 2C–E, 3A,B and Supplementary Figure 3). Diatoms were most abundant in spring and summer, when they dominated the LoV assemblages (51% in July, Figure 3A), with the centric taxa *Cyclotella* sp. and *Thalassiosira concaviuscula* in April, *Minutocellus polymorphus* and *Chaetoceros tenuissimus* in July, and benthic pennate diatoms (*Undatella* sp., *Cymbella* sp., *Achnanthes* sp.) in February. In the sea, diatoms were less abundant than in the LoV and were dominated both in April and July by *C. tenuissimus*, accompanied by *Thalassiosira* spp. and other single cells *Chaetoceros* spp. Dinoflagellates were the most abundant group in the sea (39% in April and November, Figure 3B) where they were mainly represented by chloroplast-bearing species such as *Heterocapsa pygmaea*, *Gymnodinium dorsalisulcum*, and *Tripos furca* across the year, *Alexandrium margalefii* and the heterotroph *Noctiluca scintillans* in April and *Alexandrium pseudogonyaulax* in July. The contribution of dinoflagellates was lower in the lagoon (<13% in November, Figure 3A), with *H. pygmaea* and *G. dorsalisulcum* dominating the group across the seasons, *Gymnodinium* sp. in November and *Protoperidinium tricingulatum* in February.

Ciliophora peaked in November at both sites with various Strobilidiidae in the LoV and *Strombidium* sp., *Pelagostrombidium neptunii* and Tintinnidae in the GoV. Ciliophora were abundant in the LoV also in April (e.g., *Strombidium* spp. and *Tintinnopsis* sp.) and in July (e.g., *Parastrombidinopsis minima* and *Parastrombidinopsis shimi*).

Differences among seasons and between the two environments were also common in other groups (Figures 3A–B). For example, Cryptophyceae (mainly *Teleaulax acuta*) were abundant in November at both sites, but *Rhodomonas* sp. only in the lagoon in April, while Syndiniales (unnamed taxa) peaked from April to November in the sea and in November-February in the lagoon. Some of the minor groups attained higher abundances in individual seasons and sites: Mamiellophyceae, mainly with *Ostreococcus mediterraneus* and *Micromonas bravo*, in the LoV in April (7.1%) and *M. commoda*, *M. bravo* and several other species in the GoV in February (5%); Trebouxiophyceae (*Picochlorum* sp.) in the LoV and in the GoV in July (16.1 and 8.1%, respectively); Dictyochophyceae (Pedinellales) in the LoV November (7.7%) and February (16.8%); Chrysophyceae in the LoV in November (8.4%).

In spite of the wide fluctuations in community composition, the diversity within the different groups (i.e., OTU numbers) remained relatively stable over time, especially for diatoms (Supplementary Figure 4). Differences in group-specific OTU numbers between the two habitats were also less marked compared to abundance data (i.e., reads) and mainly consisted of a higher diversity of dinoflagellates in the GoV. Besides Syndiniales, a high OTUs diversity in individual samples was shown by parasitic and heterotrophic groups such as Cercozoa, Labyrinthulea, Oomycota, and Bicoecea, which covered up to 18.8% of the total OTU numbers in the LoV.

**FIGURE 4:**
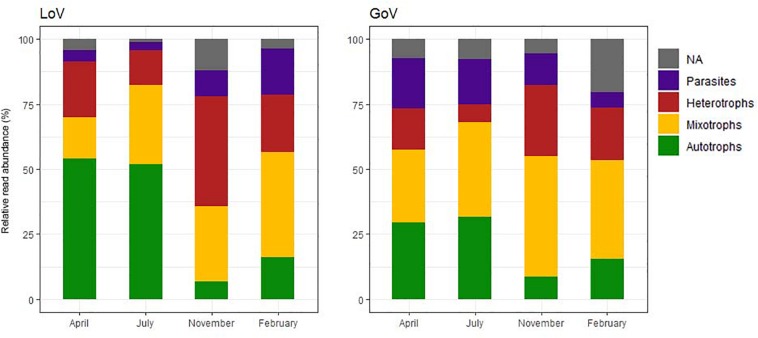
Temporal variations of the relative abundance of the trophic groups in the Lagoon (LoV) and in the Gulf of Venice (GoV) based on the whole normalized dataset (NA, not assigned).

Among the chloroplast-bearing taxa, the autotrophs (diatoms and Mamiellophyceae) prevailed in April–July while the mixotrophs (dinoflagellates and other groups) were more abundant in November–February (Figure 4), the difference across the year being more evident in the LoV. Heterotrophs (mainly ciliates, Picozoa, *Paraphysomonas* spp. and MAST) attained the highest relative abundance (42.2% in the LoV) in November. Parasites showed an opposite trend in the two environments, with the highest levels in April (19.2%, Syndiniales and Oomycota) and July (17.2%, Syndiniales) in the GoV and in February in the LoV (17.8%, Syndiniales and Labyrinthulea) (Supplementary Material 2, trophic level normalized dataset).

The α-diversity (richness and H index) was rather similar in the two sites, as both overall and monthly values (Supplementary Table 2), with a peak in April (GoV = 1362; LoV = 1376, H > 3.70). LoV samples in July had the lowest number of OTUs (826) and the lowest H (2.92), while in the GoV the minimum richness in February (757) was accompanied by a high H (4.14).

All groups were present in both the LoV and GoV, but some minor groups showed more than 90% of the reads in the lagoon (Bicoecea, Perkinsea, and Pirsonia) or in the sea (Radiozoa) (Figure 2C). The two environments shared, 96% of the total reads of the normalized dataset which however constituted only 26% of the total OTUs (1.168). Indeed, a high number of OTUs were exclusive of the LoV or the GoV (1,691 and 1,675, respectively) but they included only 1.6–1.8% of the reads of the normalized dataset (Supplementary Figure 5).

**FIGURE 5:**
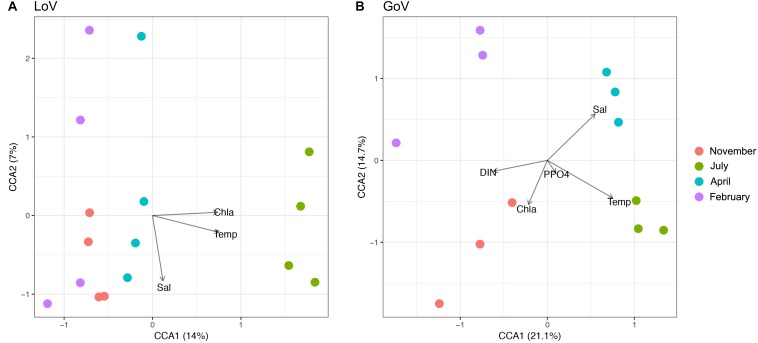
Canonical Correspondence Analysis (CCA) based on the normalized dataset. Biplot of environmental parameters: **(A)** Lagoon of Venice (LoV) community (*p* < 0.01), **(B)** Gulf of Venice (GoV) community (*p* < 0.05). Sal, salinity; Temp, temperature; DIN, Dissolved Inorganic Nitrogen; P-PO_4_, orthophosphates; Chl *a*, chlorophyll *a*.

In multivariate analyses, nMDS showed a clear separation of the community structure between the two ecosystems (Supplementary Figure 6), while CCA identified temperature and salinity as statistically significant variables (*P* < 0.001), followed by Chl *a*, Si-SiO_4_, and DIN (*P* < 0.05) (Supplementary Figure 7). For the whole area, the first two canonical axes explained 17% of the total variance (Supplementary Figure 7). The marine and lagoon samples were separated along the salinity gradient while all samples were spread along the temperature gradient. In the LoV (Figure 5A), the first two canonical axes explained 21% of the total variance, to which only temperature made significant contributions (*P* < 0.001). In the GoV (Figure 5B) the first two canonical axes explained 35% of the total variance. Temperature and salinity made significant contributions to the variance (*P* < 0.001), followed by DIN, P-PO_4_, and Chl *a* (*P* < 0.01).

#### Community Composition: Generalists vs. Specialists

**FIGURE 6:**
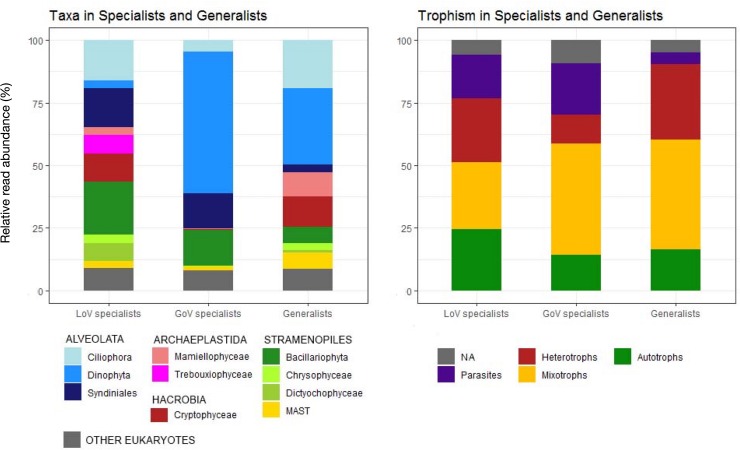
Taxonomic composition and trophic level distribution of generalists and Lagoon (LoV) and Gulf of Venice (GoV) specialists based on the OTU classification obtained with the CLAM tests (*K* = 2/3, *p* < 0.005). Normalized data from two stations in the LoV (St2 and St5) and two in the GoV (PTF and PELL), see Supplementary Material 3.

The CLAM test conducted on 2 LoV and 2 GoV stations identified 3657 OTUs (0.6% of total reads) that could not be classified with statistical confidence (Supplementary Material 3, clam dataset). Of the classified OTUs, almost 80% were “specialists,” i.e., taxa with a preference for one of the two environments (Figure 6). LoV specialists (541 OTUs) and GoV specialists (851 OTUs), however, were not always exclusive of one of the two environments. A lower number of OTUs (355) were “generalists,” i.e., with no clear preference for any of the two environments (Supplementary Material 3, clam dataset).

The taxonomic composition of the LoV and GoV specialists reflected that of the total community of the samples selected for this analysis (Figure 6 and Supplementary Figure 8), yet with an even higher contribution of Dinophyceae to the GoV specialists (412 OTUs, 56.5% of the reads) compared with their minor contribution (67 OTUs, 3.1%) to the LoV specialists. *Heterocapsa minima* represented more than a half (56.07%) of the GoV-specialist dinophycean reads, whereas a few other taxa (*Noctiluca scintillans*, *Alexandrium margalefii*, and *Tripos furca*, along with an unknown species) hardly attained more than 1% of the total (Supplementary Material 3, clam dataset). A long list of still less abundant taxa represented the remnant 408 GoV-specialist Dinophyceae OTUs. Bacillariophyta (34 OTUs) were only 14.27% of the GoV specialist reads, and were represented mainly by *Chaetoceros tenuissimus* (8.78 of the total LOV specialist reads), *Thalassiosira profunda* (2.65%), *C. muelleri*, and *C. socialis*, the latter two with less than 1% of the GoV specialist reads. An unknown Syndiniales was also quite abundant (5.45%) among the GoV specialists.

In the LoV, the most abundant and diversified specialists were Bacillariophyta (62 OTUs, 21.17% of the LoV specialist reads), of which most were *Cyclotella* sp. (43.69%) and *Minutocellus polymorphus* (35.86%), followed by *Thalassiosira* spp. (12.08%) and by a quite rich list of ca. 30 benthic araphid and raphid diatoms. A wide variety and relatively high abundance of Ciliophora (85 OTUs, 16.03% of reads) was another distinctive feature of the LoV compared to the GoV specialists. Other abundant groups of the LoV specialists were Syndiniales (157 OTUs, 15.48% of the total LoV specialists reads), followed by Cryptophyceae (11.34%, mainly *Teleaulax acuta* and *Rhodomonas* sp.) and Trebouxiophyceae (7.50%, mainly *Picochlorum* spp.).

The most abundant generalists were Dinophyceae (80% represented by *Gymnodinium dorsalisulcum*), several species of Ciliophora, Cryptophyceae, Mamiellophyceae (*Micromonas* and *Ostreococcus* spp.) and MAST.

Differences in composition were also reflected in differences in the trophic structure of the two environments, with mixotrophs more widely represented in the GoV specialists and autotrophs and heterotrophs more abundant in the LoV specialists (Figure 6). Parasites were more represented in specialists than in generalists, whereas the opposite occurred for heterotrophs.

#### Network Analysis

**FIGURE 7:**
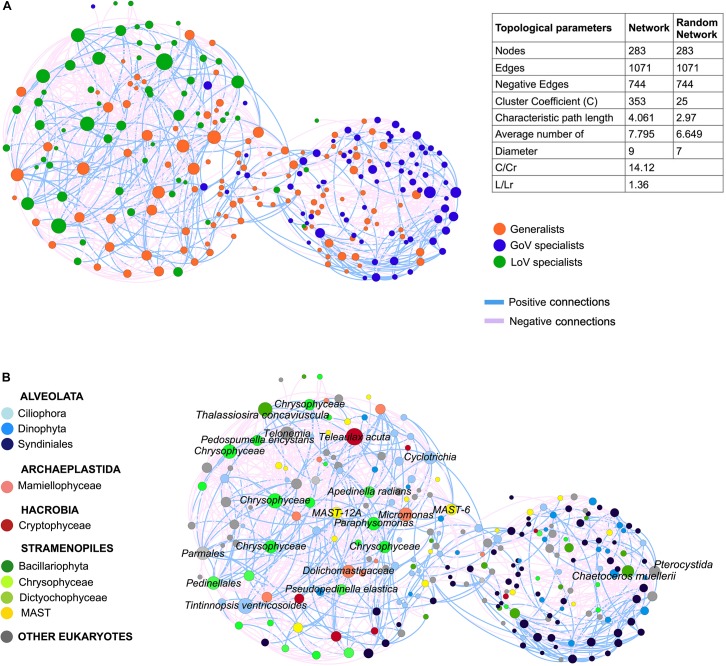
Network of the normalized dataset (28 samples, OTUs > 50 reads) based on MIC-MINE algorithm. The size of nodes corresponds to the degree value (i.e., number of connections): **(A)** Network annotation (color of nodes) based on clam test prediction (generalists and specialists) and table of statistics for original and random network (C, clustering coefficient; L, characteristic path length; Cr, clustering coefficient of the random networks; Lr, characteristic path length of the random network); **(B)** Network annotation (color of nodes) based on the taxonomic affiliation and taxonomic details (name of nodes) for the 22 hubs.

The MIC-MINE analysis produced a network with 283 nodes and 1071 edges. The clustering coefficient (C) and the characteristic path length (L) in the original network (C = 0.353, L = 4.061) were higher than the average from the random networks (Cr = 0.025, Lr = 2.97). The annotation of nodes with the generalist/specialist classification identified a clear separation between LoV and GoV, along with a lower degree of connections among GoV specialists compared to LoV ones (Figure 7). A total of 22 nodes showed degree values between 39 and 20 (hubs); among them. 16 were annotated as LoV specialists, two as GoV specialists and four as generalists (Supplementary Material 4, network annotation). The 16 LoV specialist hubs included taxa such as Ciliophora (*Tintinnopsis* sp. and *Cyclotrichia* sp.), *Telonemia*, Dictyochophyceae (including *Apedinella radians* and undetermined species), Chrysophyceae (including the terrestrial species *Pedospumella encystans* and undetermined species), Cryptophyceae and Bacillariophyta (*Thalassiosira* spp.). The two GoV specialist hubs comprised Bacillariophyta (*Chaetoceros muellerii*) and one Centroheliozoa (Pterocystida) OTU. The four generalist hubs were represented by taxa belonging to the Mamiellophyceae, Chrysophyceae, Dictyochophyceae and MAST.

In addition to the higher number of hubs. the number of connections was higher in the lagoon than in the sea (571 vs. 263 connections), with a quite high number of negative interactions (more than 60%) which were more densely found between the LoV nodes.

## Discussion

The analysis of the V4 hypervariable region of the 18S rRNA gene in the Lagoon and Gulf of Venice on four dates provides the first detailed overview of planktonic protists in an area where morphology-based studies on these communities date back to XIX century. During the last decades, ca 300 microalgal (Bernardi Aubry et al., 2012, 2013, 2017) and a few microzooplankton taxa (Bandelj et al., 2008) were identified in light microscopy, which is a much lower number compared to the more than 7,000 OTUs recorded in the present study. Considering that our dataset only included four sampling dates, the actual diversity of planktonic protists is likely even greater than the one observed in this study. This result is common to other metabarcoding studies in LTER areas (e.g., Piredda et al., 2017) and is not unexpected. Traditionally whole groups, mainly heterotrophs, parasites, and picoeukaryotes, have received scarce attention if any, while other groups are difficult to identify with light microscopy or even unseen in fixed material (e.g., small-sized flagellates). Results of this study clearly show that all these groups are well represented with several distinct species in both the LoV and the GoV. For example, several green picoplanktonic coccoids or flagellates, not reported before from neither the LoV nor the GoV (e.g., *Picochlorum*, *Micromonas*, *Ostreococcus*, and *Bathycoccus*), were abundant in the metabarcoding dataset.

Also in the case of diatoms, which instead have been studied based on morphology (Bernardi Aubry et al., 2004, 2012, 2013, 2017; Cabrini et al., 2012; Marić et al., 2012), the present study reveals many more taxa than those known for the area, likely because of the capability of the molecular approach to detect rare species and to resolve cryptic diversity, which is widespread also in this taxonomic group (Sarno et al., 2007; Lundholm et al., 2012; Percopo et al., 2016). Benthic, tychoplanktonic species (e.g., *Nitzschia* spp., *Navicula* spp., and *Amphora* spp.), reported as typical lagoon inhabitants (Bernardi Aubry et al., 2013), were quite diversified in the metabarcoding results but with low read numbers, probably due to the limited time span or because they were not identified due to the well-known lack of reference sequences for benthic diatoms (Piredda et al., 2018).

Among dinoflagellates, particularly interesting was the detection of several potentially toxic species of the genera *Alexandrium* and *Azadinium* and of *Gambierdiscus australes*, as well as, of rare taxa such as the dinoflagellate *Posoniella tricarinelloides*, a species only found in fossil records until its recent recovery from cyst germination (Gu et al., 2013). Yet the attribution of reads to a named species should be taken with caution even when the environmental reads fully match the reference sequences, because distinct species may share the same V4 sequence (e.g., in several diatoms and dinophycean species), and therefore their sequence-based identification is not certain (Luddington et al., 2012).

A common feature in the two environments was the temporal variability signal, with the dominance of autotrophic forms, mainly diatoms, especially in spring and summer and the alternation of different taxa across the year. Similarly, signals of seasonality and shift of protist community composition along the year emerged clearly from several previous studies from marine ecosystem in the Mediterranean Sea (Piredda et al., 2017; Giner et al., 2019), and English Channel (Genitsaris et al., 2016; Lambert et al., 2019). However, the relative abundance of groups and species and their temporal variability were clearly different between the lagoon and the sea.

The difference of salinity between the LoV and the external coastal Adriatic waters is low and should not constitute a barrier for marine microeukaryotes. Hence it is not surprising that marine species are predominant in the whole dataset and that most species thriving in the lagoon also inhabit the external coastal waters, and viceversa, like in the case of other mixohaline lagoons of the Italian coasts (e.g., Sarno et al., 1993). Some of these shared species can also attain comparably high abundance in both sites (i.e., the generalists in the CLAM analysis). However, high proportion of specialists with a differential distribution in the two areas indicates specific ecological conditions that structure the local communities in different ways. Among LoV specialists are several species commonly found in lagoons or estuarine environments, such as *Teleaulax acuta*, *Picochlorum* spp., *Cyclotella* sp. and *Minutocellus polymorphus* (Sarno et al., 1993; Bérard-Therriault et al., 1999; Melo et al., 2010; Guiry and Guiry, 2019), while the majority of GoV specialists are Dinophyceae that have a considerably lower importance in the LoV.

In addition to the dominant specialists, also less abundant taxa were distributed preferentially or even exclusively in the LoV or in the GoV. The latter was the case of a large part of the OTUs that included only a minor part of the total reads. This large diversity allocated in the rare OTUs (Avg: 6 reads per OTU, ca. 0.001% of the total dataset) points at a common structure in protist communities where a limited number of species play the main functional roles and the rare ones constitute the biodiversity reservoir (Jousset et al., 2017). The finding of a high number of rare OTUs exclusively in the LoV or in the GoV could depend on their abundance at the boundary of the detection limit (Logares et al., 2015), which makes their differential distribution not completely sound, but could also indicate a specific composition of the biodiversity reservoir of the two ecosystems, in line with the biogeographic signal found in the rare component of metabarcoding datasets at a larger, European scale (Logares et al., 2014, 2015).

The spatial differences in plankton communities emerging from the present study confirm what was known from previous years and studies (Acri et al., 2004; Bernardi Aubry et al., 2004, 2013) and can therefore be considered quite typical of the area. Interestingly bacterial communities analyzed with the same approach also showed both spatial and temporal differences in the GoV and LoV, along with a prevalent role of seasonality in shaping prokaryotic assemblages (Quero et al., 2017). In the same study, prokaryotic diversity was much higher in the lagoon due to the contribution of microbial communities from both the watershed and coastal waters in that site. This pattern was less evident in our data, where various diversity indices do not show significant differences between the two environments.

The relationship between the LoV and GoV is further confirmed by the ecological network, where specialists of each ecosystem identified two distinct but connected sub-networks, with a high number of connections of which many were negative. High amount of negative interactions is generally interpreted as the result of functional heterogeneity, direct competition for limiting resources or interactions like predator-prey relations or allelopathy (Long and Azam, 2001). However, positive or negative ecological interactions may simply reflect co-occurrence or non-coexistence patterns among populations (Hernández-Ruiz et al., 2018). Particularly in this study negative interactions may just derive from the spatial and temporal ecological niche partitioning, which matches all other results that point to different communities characterizing the two ecosystems in the different seasons. Positive interactions, indicating groups of organisms having similar, complementary or cooperative functions or activities, but also common preferred environmental conditions (Fuhrman and Steele, 2008; Eiler et al., 2012), were less evident among the communities of the area.

In the interpretation of network properties, the ecological role of hub species is still unclear. In several studies, hubs are often proposed to be critical or keystone components for network (Peura et al., 2015; Comte et al., 2016; Hernández-Ruiz et al., 2018) but a recent study has also demonstrated that known keystone species do not necessarily result in detectable signals in co-occurrence networks (Freilich et al., 2018). In our study, most hubs were not dominant in abundance, suggesting that scarcely abundant but highly connected OTUs could play an important role in network dynamic and stability not only in marine communities (Xue et al., 2018) but also in the lagoon habitat.

The higher number of hubs ascribed to LoV specialists and the higher number of connections suggest a higher complexity and species inter-dependence among planktonic protist communities in the lagoon ecosystem. Interactions among species would frequently be enhanced in lagoons as well as in harbors and other semi-enclosed areas, possibly due to the proximity to the bottom and to the benthic vegetation, or to any other effects deriving from being confined environments (Margalef, 1969; Guelorget and Perthuisot, 1983; Perez-Ruzafa and Marcos, 1992, 1993; Sarno et al., 1993; Kjerfve, 1994) and to the degree of connectivity with the sea (Ghezzo et al., 2015; Perez-Ruzafa et al., 2019). Different drivers are probably involved in shaping protist communities in the GoV, where the number of connections among specialist hubs is considerably lower. Considering also the higher variance explained by environmental variables, a higher influence of abiotic factors seems to be at stake in the GoV. This picture would also be reflected in the annual phytoplankton cycle of the GoV, which is more irregular than in the lagoon, with minor peaks alternating in spring and summer, due to the combination of nutrient depletion and sporadic nutrient inputs (Bernardi Aubry et al., 2006, 2012; Socal et al., 2008).

## Conclusion

Long Term Ecological Research sites constitute inspiring places, where long term observations stimulate a wide range of specific research activities (Peters, 2010) that provide, in turn, precious tools for interpreting long term data, thus increasing their informative value (Zingone et al., 2019). This is the case for metabarcoding studies which are particularly valuable at LTER sites, where the existing background ecological knowledge allows optimizing both the arrangement of the molecular research and the interpretation of its results (Davies et al., 2014; Stern et al., 2018).

At the LTER-Italy sites of the Lagoon and Gulf of Venice, two strictly coupled transitional and marine ecosystems, long-term studies conducted with light microscopy have so far provided the basis for the current understanding of the spatial and temporal variability of plankton in the area. This investigation of the whole protistan community based on the V4-18S rRNA, which is the first in the area, has largely increased the knowledge about protist diversity not only for groups that have traditionally been neglected (mainly heterotrophs, parasites, and picoeukaryotes), but also for the main phytoplankton taxa studied in the long term with morphology-based approaches (e.g., diatoms and dinoflagellates). The molecular database obtained in this study will be a reference for future studies and foster further taxonomic explorations, which will result in an improvement of the quality of the long term dataset.

Further, metabarcoding results clearly indicate spatial differences in the structure of the protistan communities and their changes over the time in an area where habitat heterogeneity and connection coexist. Despite the presence of the most abundant taxa at both sites, their relative contribution and temporal variability are indeed different between the two environments, while the rare taxa are also exclusive of one of the two environments in many cases. Indeed, specific features of the lagoon and the sea communities clearly emerge from all our results. In these so close ecosystems, environmental heterogeneity appears strong enough to allow for ecological segregation, despite no clear barrier to dispersal processes among local protist communities.

Finally, a higher degree of species inter-dependence among planktonic protists has emerged in the lagoon compared to the adjacent coastal waters, suggesting that different drivers are at play in shaping communities in the two ecosystems, with a prevalence of biotic interactions in the lagoon and a higher influence of abiotic factors in the sea.

Overall, these results provide a starting point and a sound motivation for extending the analysis to multiple years also including other components of the community, e.g., bacteria and metazoans, in order to deepen the knowledge of the seasonal patterns and the biotic interactions that is needed for monitoring and managing changes in these LTER sites.

## Data Availability Statement

All datasets generated/analyzed for this study are included in the article/Supplementary Material.

## Author Contributions

SA, GQ, FB, AP, and AZ designed the experiment. SA, SF, FB, and MB provided samples and environmental data. SA, RP, GQ, and SF performed the DNA extraction. GQ and RP performed the bioinformatic and statistical analyses. SA, GQ, RP, SF, FB, AP, and AZ contributed to the interpretation of the results. SA and RP wrote the first draft of the manuscript. SA, RP, GQ, FB, SF, AZ, and AP finished the manuscript. All authors contributed to the revised version, and read and approved the final version of the manuscript.

## Conflict of Interest

The authors declare that the research was conducted in the absence of any commercial or financial relationships that could be construed as a potential conflict of interest.

## Footnotes

1 www.lteritalia.it

2 http://www.ismar.cnr.it/infrastructures/piattaforma-acqua-alta

3 https://www.ncbi.nlm.nih.gov/sra

4 https://www.ncbi.nlm.nih.gov/bioproject/PRJNA576330

5 https://github.com/pr2database/pr2database

6 http://www.exploredata.net
