# Supplementary material for: Habitat Heterogeneity and Connectivity: Effects on the Planktonic Protist Community Structure at Two Adjacent Coastal Sites (the Lagoon and the Gulf of Venice, Northern Adriatic Sea, Italy) Revealed by Metabarcoding
Source: Front Microbiol. 2019 Nov 26;10:2736. doi: 10.3389/fmicb.2019.02736 (PMC6988810; doi:10.3389/fmicb.2019.02736)
Supplement: Supplementary file 2 [file Data_Sheet_1.pdf]

Supplementary Material

Supplementary Table 1. Averages and standard deviations of the abiotic and biotic variables in the LoV and GoV on each sampling date of this study. The seasonal means of the variables in the previous five years (from 2011 to 2015) are also reported for comparison.

Station	Month	Temperature (°C)	Salinity (PSU)	DIN (µM)	$Si\text{-}SiO_4$ (µM)	$P\text{-}PO_4$ (µM)	Chl *a* (µg/l)
LoV	April	23.9±6.2	29.0±3.3	18.7±6.6	25.0±14.5	0.5±0.5	2.8±2.6
	July	24.3±7.5	28.8±2.9	25.9±12.4	34.5±19.0	0.9±0.6	4.8±4.4
	Nov	14.9±5.8	29.7±2.2	43.7±21.0	38.7±22.4	1.1±0.7	0.5±0.3
	Feb	12.9±5.9	28.9±4.7	28.5±21.5	17.8±18.8	0.7±0.9	1.2±0.9
GoV	April	20.5±4.4	33.5±2.5	9.6±8.7	5.5±4.1	0.2±0.3	4.0±6.6
	July	25.6±1.5	34.8±0.7	5.4±3.4	7.4±4.8	0.2±0.1	1.1±0.4
	Nov	13.3±2.8	33.5±3.1	21.8±15.4	14.6±6.4	0.2±0.1	1.7±1.0
	Feb	9.0±3.1	34.4±1.8	15.4±7.0	6.2±4.9	0.3±0.30	1.3±0.8
LoV (2011-2015)	Spring	23.1±4.3	26.2±4.2	35.8±27.4	33.7±23.3	0.9±1.1	3.5±3.7
	Summer	27.5±2.8	29.4±3.7	16.7±14.2	26.1±16.1	0.9±1.1	10.2±16.3
	Autumn	16.1±5.9	27.9±4.5	44.9±27.6	39.6±31.2	1.0±2.1	1.0±1.3
	Winter	11.0±4.0	26.0±5.2	63.6±41.4	28.9±19.6	0.5±0.4	2.36±3.80
GoV (2011-2015)	Spring	19.2±3.4	32.2±3.0	16.6±14.4	8.2±7.01	0.3±1.1	2.3±2.1
	Summer	25.5±1.7	33.0±2.2	8.7±7.1	6.2±3.4	0.1±0.1	1.6±1.7
	Autumn	14.7±5.0	32.5±3.9	18.9±12.1	11.8±9.4	0.1±0.1	1.4±1.2
	Winter	9.2±2.3	33.3±3.0	25.3±16.8	12.2±7.4	0.1±0.1	1.3±0.8

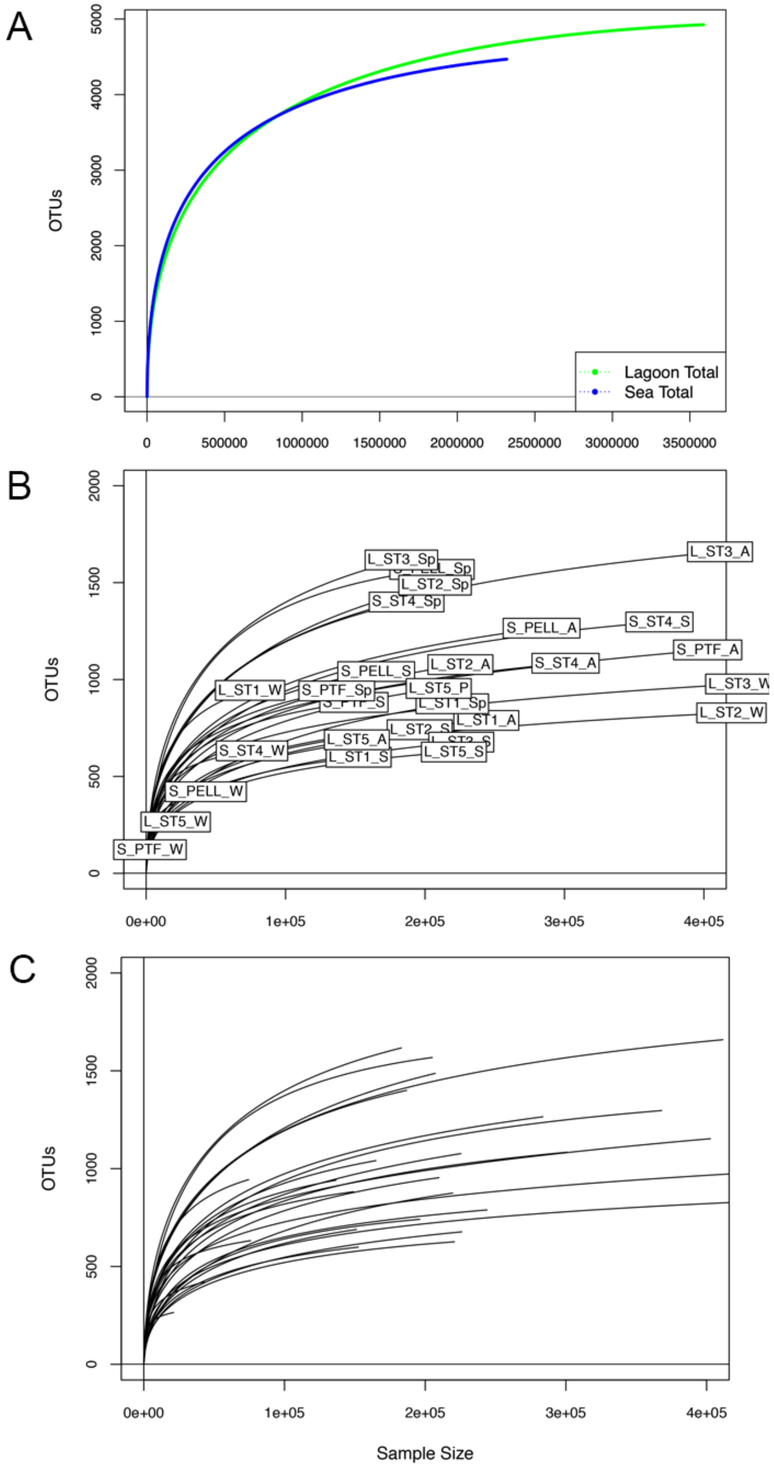

Supplementary Figure 1. Rarefaction curves showing the number of OTUs (97%) compared to the number of reads (sample size) observed in the total dataset. Panel A: rarefaction curves of the total lagoon and total sea samples. Panel B: rarefaction curves of each of the 28 samples. In the labels, the initial letters L and S indicate LoV and GoV stations, respectively, while the final letters are the initials of the seasons. Panel C: rarefaction curves as in B, without labels for a clearer presentation of the data.

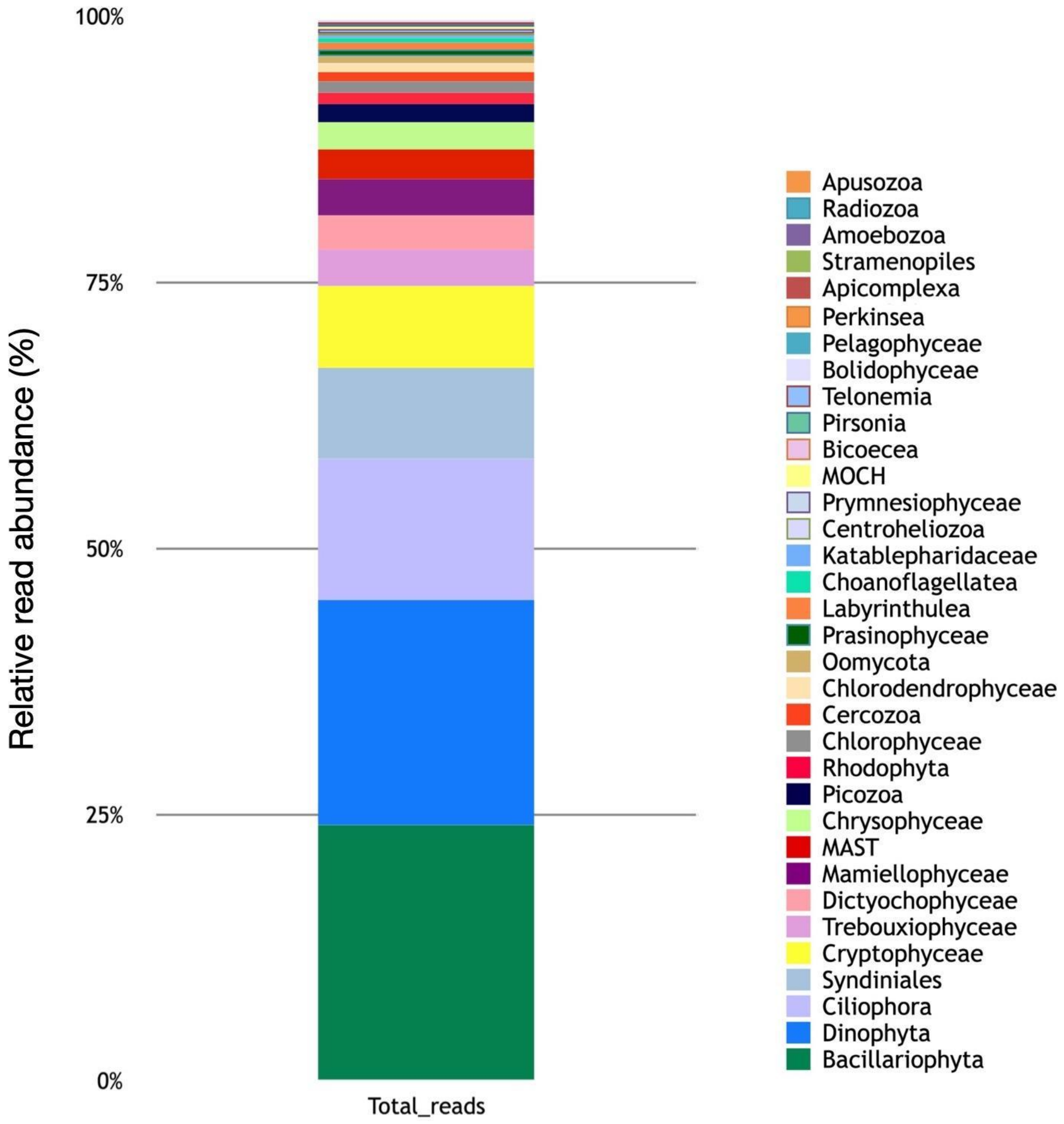

Supplementary Figure 2. Taxonomic diversity of the normalized dataset as the percentage of reads belonging to the 34 high-level taxonomic groups.

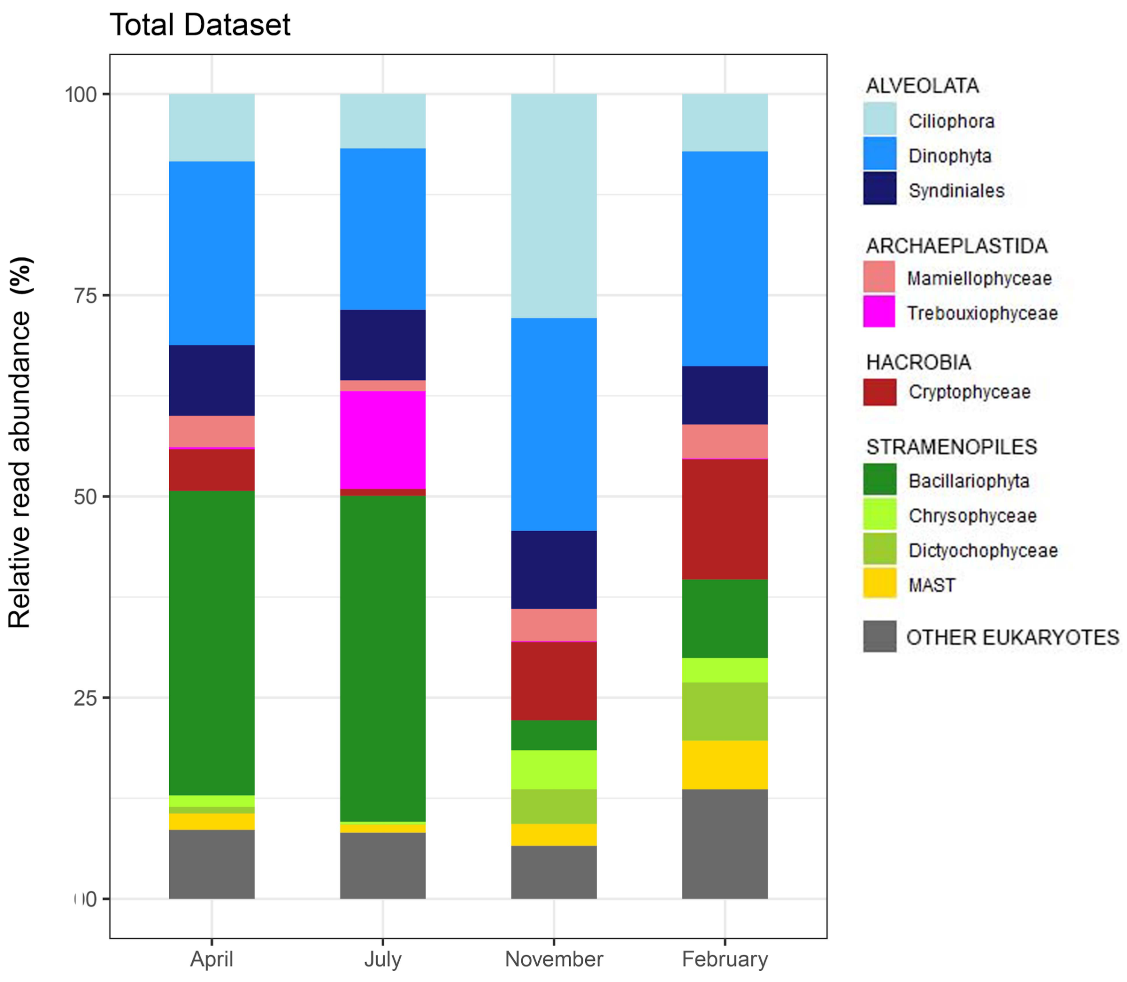

Supplementary Figure 3. Temporal variations of the protist community composition in the total normalized dataset. Taxa with relative abundances <5% within each month were grouped into "Other Eukaryotes".

Supplementary Table 2. Alpha-diversity in the LoV and GoV described by richness (number of observed OTUs) and Shannon index (H).

	**Habitat**		**LoV**				**GoV**			
	**LoV**	**GoV**	**Apr**	**Jul**	**Nov**	**Feb**	**Apr**	**Jul**	**Nov**	**Feb**
Richness	2859	2843	1376	826	1118	1133	1362	1020	1006	757
H	4.52	4.45	3.95	2.92	3.72	4.16	3.79	3.78	3.49	4.14

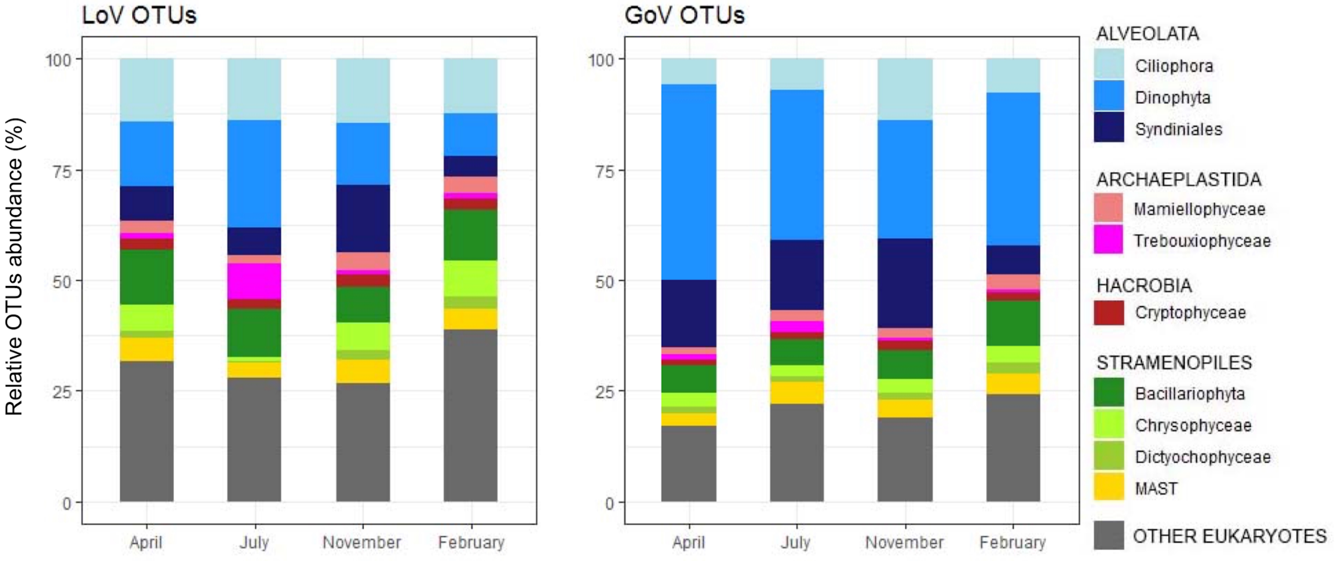

Supplementary Figure 4. Temporal variations of of the diversity (as OTU numbers) of the main protist groups in the LoV and the GoV, based on the normalized dataset.

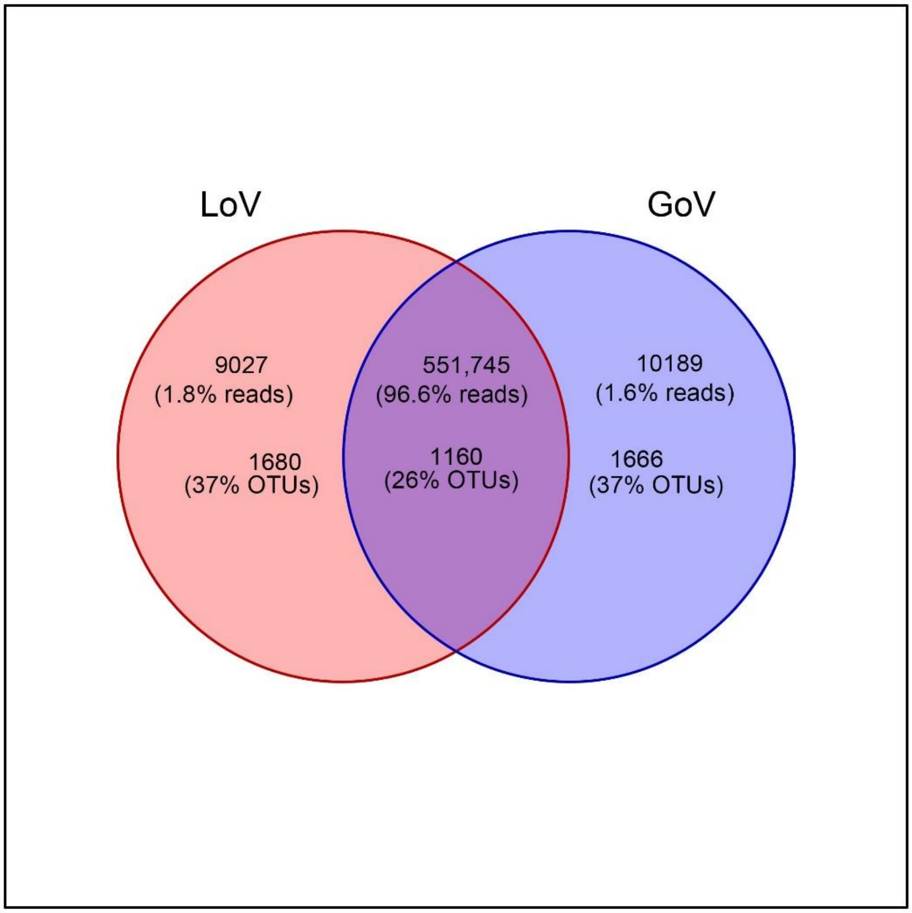

Supplementary Figure 5. Venn Diagram showing the number and % of OTUs and reads found in LoV and GoV, and those shared between the two environments (based on the normalized dataset).

Supplementary Material

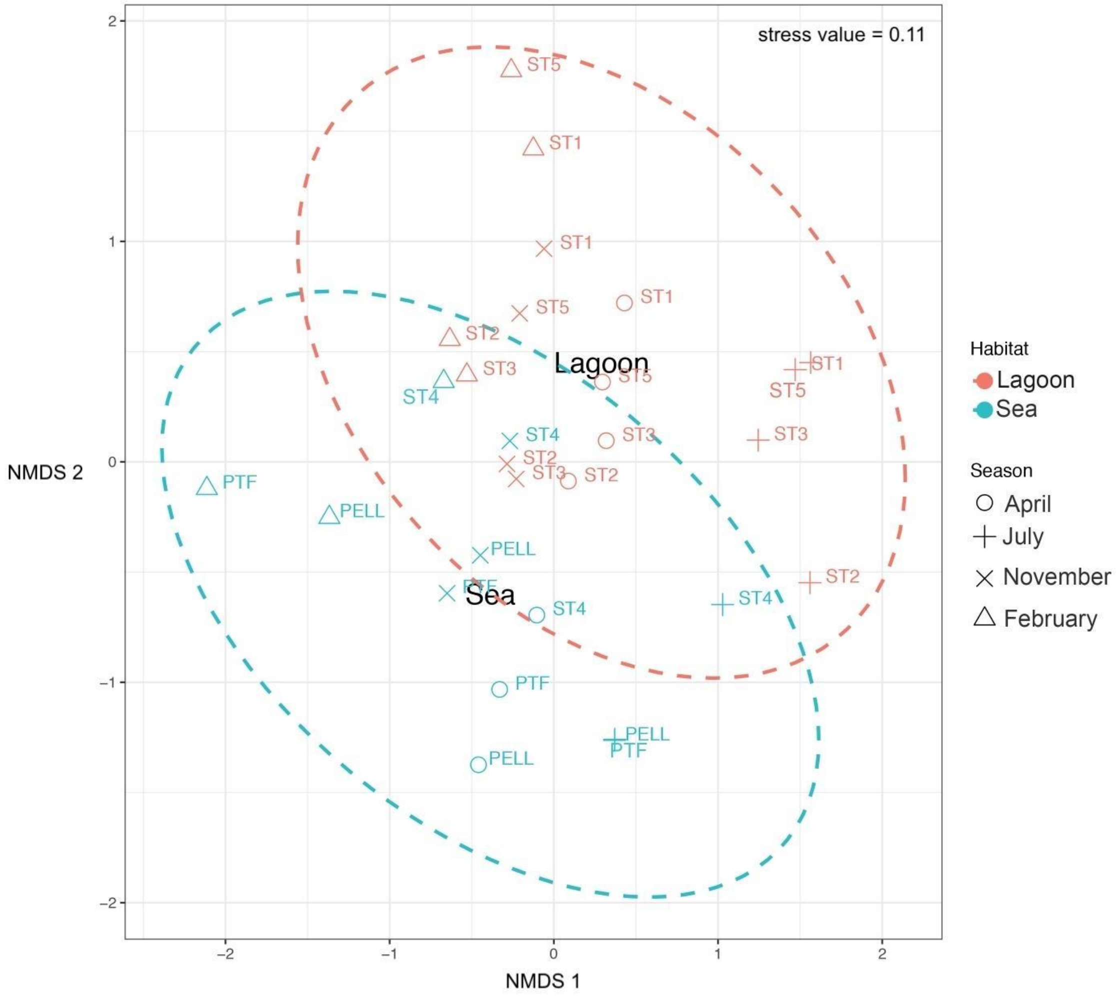

Supplementary Figure 6. Seasonal Non-metric MDS (nMDS) ordination of protist community composition in the Lagoon and Gulf of Venice stations, based on the Bray–Curtis dissimilarity matrix calculated on the OTU table. Stress value = 0.11.

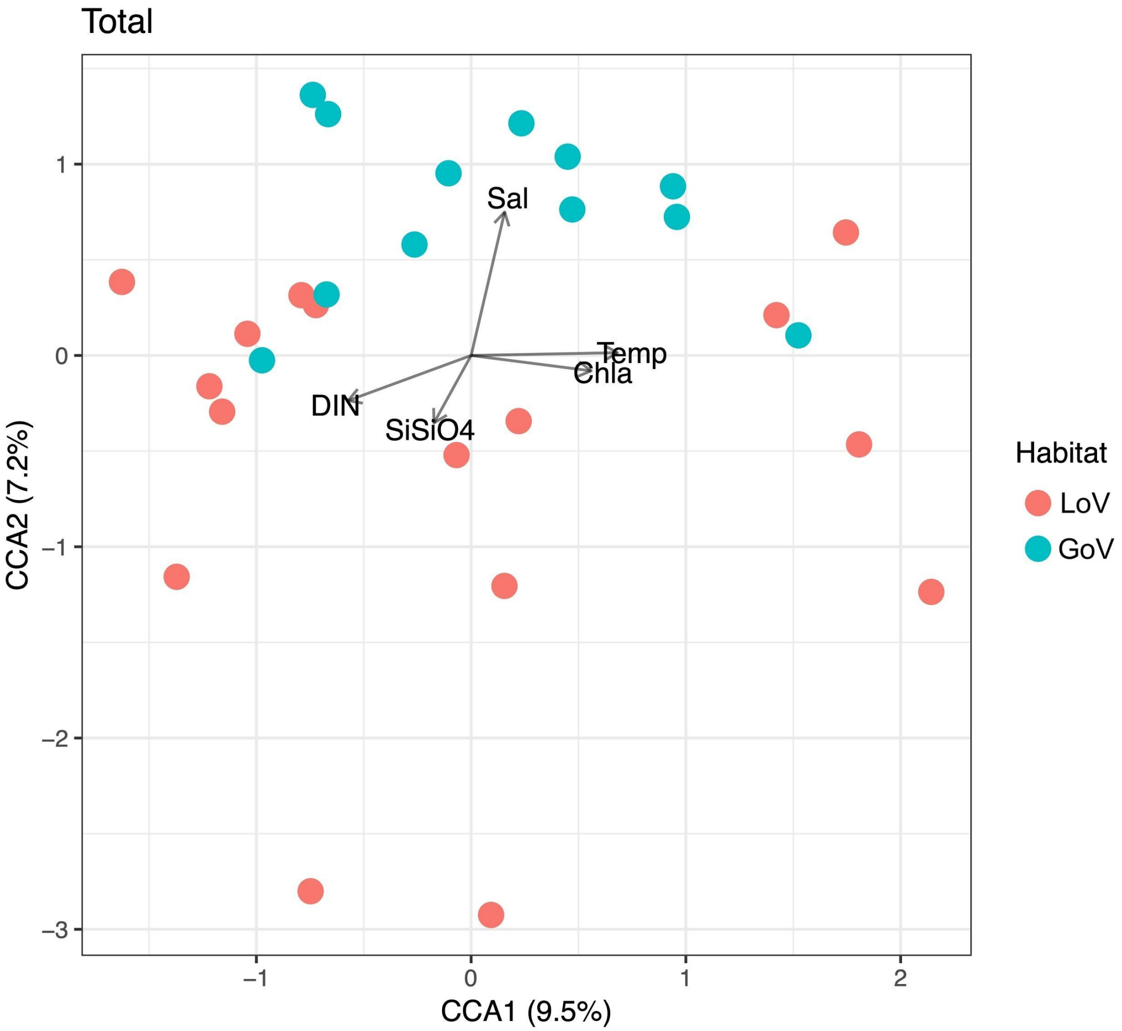

Supplementary Figure 7. Canonical Correspondence Analysis (CCA). Biplot of environmental parameters of the total protistan community ($p<0.05$). Sal = salinity; Temp = temperature; DIN = Dissolved Inorganic Nitrogen; Si-SO4 = orthosilicates; Chl a = chlorophyll a.

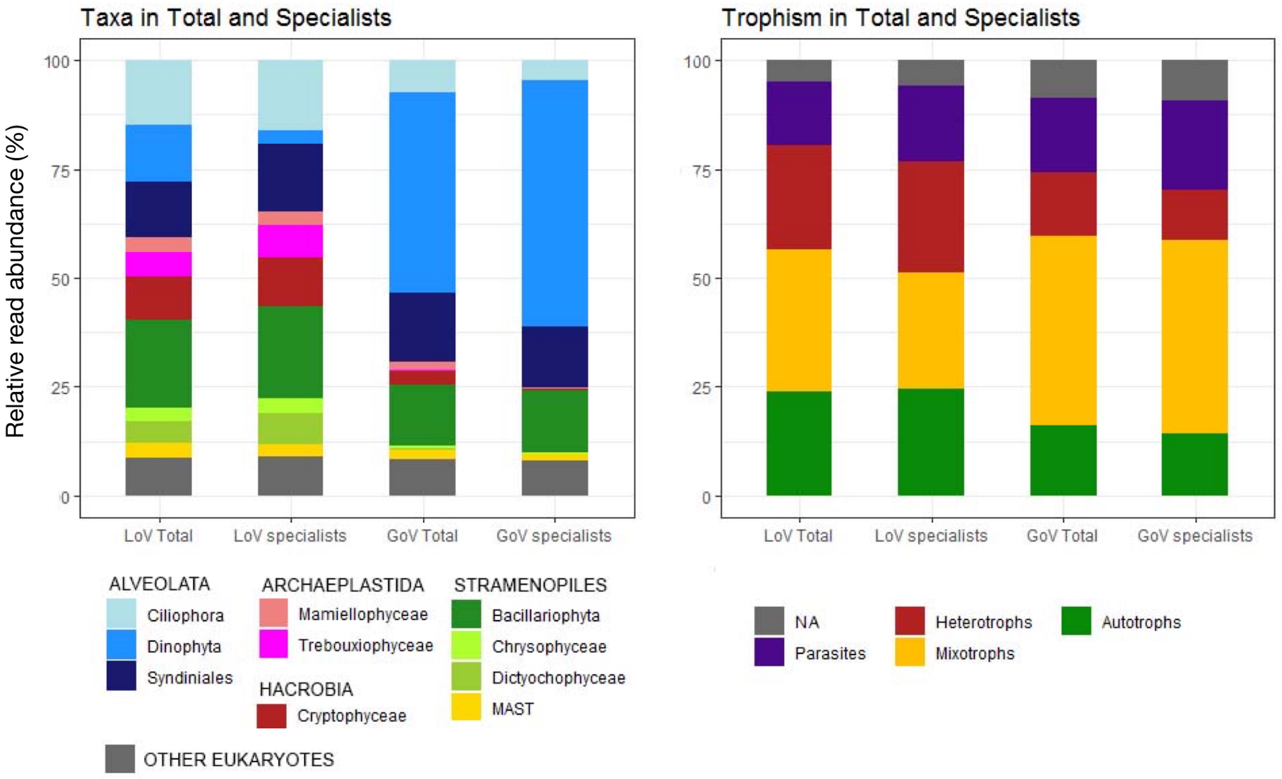

Supplementary Figure 8. Taxa and trophic level distribution in the GoV and LoV specialists compared to their distribution in the total dataset of the 4 stations selected for the CLAM tests.

Joined file:

Supplementary Material.xls*:* Including the following sheets: Supplementary Material 1: *Taxonomic assignment;* Supplementary Material 2: *Normalized dataset with trophic level;* Supplementary Material 3: *clam dataset.* Supplementary Material 4: *network annotation*
